# Solving the mystery of Obake rice in Africa: population structure analyses of *Oryza longistaminata* reveal three genetic groups and evidence of both recent and ancient introgression with *O. sativa*

**DOI:** 10.3389/fpls.2023.1278196

**Published:** 2023-11-15

**Authors:** Marlee R. Labroo, Lindsay V. Clark, Shilai Zhang, Fengyi Hu, Dayun Tao, Ruaraidh Sackville Hamilton, Erik J. Sacks

**Affiliations:** ^1^Department of Crop Sciences, University of Illinois at Urbana-Champaign, Urbana, IL, United States; ^2^School of Agriculture, Research Center for Perennial Rice Engineering and Technology in Yunnan, Yunnan University, Kunming, China; ^3^Yunnan Seed Laboratory & Yunnan Key Laboratory for Rice Genetic Improvement, Food Crops Research Institute, Yunnan Academy of Agricultural Sciences (YAAS), Kunming, China; ^4^T.T. Chang Genetic Resources Center, International Rice Research Institute (IRRI), Los Baños, Philippines; ^5^CGIAR Genebank Initiative, Salisbury, United Kingdom

**Keywords:** *Oryza longistaminata*, *Oryza sativa* (domesticated Asian rice), *Oryza glaberrima* (domesticated African rice), interspecific hybridization, crop wild relatives (CWR), AA genome, germplasm conservation, population genetics

## Abstract

The undomesticated rice relative *Oryza longistaminata* is a valuable genetic resource for the improvement of the domesticated Asian rice, *Oryza sativa*. To facilitate the conservation, management, and use of *O. longistaminata* germplasm, we sought to quantify the population structure and diversity of this species across its geographic range, which includes most of sub-Saharan Africa, and to determine phylogenetic relationships to other AA-genome species of rice present in Africa, including the prevalence of interspecific hybridization between *O. longistaminata* and *O. sativa*. Though past plant breeding efforts to introgress genes from *O. longistaminata* have improved biotic stress resistance, ratooning ability, and yield in *O. sativa*, progress has been limited by substantial breeding barriers. Nevertheless, despite the strong breeding barriers observed by plant breeders who have attempted this interspecific cross, there have been multiple reports of spontaneous hybrids of *O. sativa* and *O. longistaminata* (aka “Obake”) obtained from natural populations in Africa. However, the frequency and extent of such natural introgressions and their effect on the evolution of *O. longistaminata* had not been previously investigated. We studied 190 *O. longistaminata* accessions, primarily from the International Rice Research Institute genebank collection, along with 309 *O. sativa*, 25 *Oryza barthii*, and 83 *Oryza glaberrima* control outgroups, and 17 control interspecific *O. sativa*/*O. longistaminata* hybrids. We analyzed the materials using 178,651 single-nucleotide polymorphisms (SNPs) and seven plastid microsatellite markers. This study identified three genetic subpopulations of *O. longistaminata*, which correspond geographically to Northwestern Africa, Pan-Africa, and Southern Africa. We confirmed that *O. longistaminata* is, perhaps counterintuitively, more closely related to the Asian species, *O. sativa*, than the African species *O. barthii* and *O. glaberrima*. We identified 19 recent spontaneous interspecific hybrid individuals between *O. sativa* and *O. longistaminata* in the germplasm sampled. Notably, the recent introgression between *O. sativa* and *O. longistaminata* has been bidirectional. Moreover, low levels of *O. sativa* alleles admixed in many predominantly *O. longistaminata* accessions suggest that introgression also occurred in the distant past, but only in Southern Africa.

## Introduction

Global consumption of Asian domesticated rice, *Oryza sativa*, is projected to rise from 439 million tons to 555 million tons between 2010 and 2035 (Seck et al., 2012). To keep pace with demand, annual rice yield growth must increase by 0.2%–0.5% (Mohanty et al., 2013). The rice wild relative *Oryza longistaminata* is a valuable genetic resource for trait improvement of Asian domesticated rice, *O. sativa* (Khush et al., 1991; Ramos et al., 2016). A perennial, cross-pollinated species native to all of sub-Saharan Africa, *O. longistaminata* is highly diverse (Bezançon et al., 1977; Kiambi et al., 2018; Kiambi et al., 2005; Melaku et al., 2013). Of the eight AA-genome species that make up the primary germplasm pool of domesticated rice, *O. longistaminata* is one of the most genetically distinct from *O. sativa* (Zhu et al., 2014; Wambugu et al., 2015). *O. longistaminata* is distantly related to the two other AA-genome species of rice indigenous to Africa—a domesticated species, *Oryza glaberrima*, and its wild progenitor, *Oryza barthii* (Zhu et al., 2014; Wambugu et al., 2015). Though past breeding efforts have introgressed valuable traits from *O. longistaminata* to domesticated Asian rice, including bacterial blight resistance, perennial habit, and floral traits conducive to outcrossing, the development of genetic resources for this understudied wild species would assist further progress (Ronald et al., 1992; Sanni et al., 2013; Atwell et al., 2014; Gichuhi et al., 2016; Zhang et al., 2017; Prahalada et al., 2021; Zhang et al., 2023). Understanding *O. longistaminata* population structure and genetic diversity based on high genome coverage across the full geographic range of this species would facilitate conservation planning, germplasm management, and improvement of domesticated rice (Wambugu et al., 2013).

Because hybridization with *O. sativa* is complicated by substantial breeding barriers (e.g., endosperm abortion, F_1_ sterility, and hybrid breakdown), manipulating the crossability between these two species could accelerate introgression efforts (Chu and Oka, 1970a; Chu and Oka, 1970b; Causse and Ghesquiere, 1991). The success rate of laboratory efforts at hybridization has varied from less than 1 in 1,000 (Ramos, IRRI, Philippines, pers. comm.) to 1 in 50 (Chu and Oka, 1970a; Kaushal and Ravi, 1998). Recovering interspecific progeny has typically required embryo rescue (Causse and Ghesquiere, 1991; Khush et al., 1991; Tao and Sripichitt, 2000; Ramos et al., 2016). In contrast to the strong breeding barriers observed in the laboratory, however, Bezançon et al. (1977); Chu and Oka (1970b); Ghesquière (1986); Kanya (2010), and Kilewa (2014) reported the occurrence of spontaneous hybrids between *O. sativa* and *O. longistaminata*, especially along the edges of rice fields in Africa. Chu and Oka (1970b) named these spontaneous hybrids between *O. sativa* and *O. longistaminata*, “Obake”, a Japanese word for a spirit that is in a state of change or transformation, which is an apropos metaphor for a hybrid swarm. However, it is unclear how frequent are such spontaneous hybrids between *O. sativa* and *O. longistaminata* in Africa and if their occurrence is only recent or a long-extant phenomenon, especially given that *O. sativa* is not native to Africa. On an applied level, identification of natural hybrids of *O. sativa* and *O. longistaminata*, including backcross generations, could facilitate introgression efforts if plant breeders can identify individuals that have *O. longistaminata* genes, but which are also highly crossable with *O. sativa*. Given the broad geographic range of *O. longistaminata*, the species’ great potential as a source of traits for improving domesticated rice, and the need for a continent-wide and genome-wide population genetics analysis, the present study was conducted to 1) characterize the population structure of *O. longistaminata* throughout its native range in sub-Saharan Africa using 190 accessions primarily from the International Rice Research Institute (IRRI) genebank, 2) quantify genetic diversity of *O. longistaminata* overall and among its genetic groups, and 3) determine if previous reports of spontaneous interspecific hybrids between *O. sativa* and *O. longistaminata* in Africa can be confirmed in the IRRI germplasm collection and to conduct an Africa-wide quantitative assessment of recent and past introgressions.

## Materials and methods

### Plant materials

We studied 190 accessions of *O. longistaminata*, collected from much of the species’ native geographic range in sub-Saharan Africa; this included all of the available and viable accessions in the IRRI genebank and one accession from Yunnan University (**Table S1**). On average, these *O. longistaminata* accessions have undergone three seed-increases in a screenhouse since acquisition by the IRRI genebank. Up to two individuals from each *O. longistaminata* accession were sampled, totaling 365 individuals. Seeds were aseptically germinated, and *O. longistaminata* plants were grown to maturity in a greenhouse in Urbana, IL. Atypical phenotypes in the *O. longistaminata* individuals were recorded (e.g., lack of rhizomes, filled grains produced by selfing, and short stature; Vaughan, 1994). Control outgroups of *O. sativa* (n = 309), *O. barthii* (n = 25), and *O. glaberrima* (n = 83) were obtained as seeds from the USDA National Plant Germplasm System or Dr. Fengyi Hu’s perennial rice breeding program at Yunnan University, or their sequence data only were accessed via the 3k Rice Genomes Project or the National Center for Biotechnology Information (NCBI) Sequence Read Archive (SRA), with one individual per accession studied for these inbreeding species (**Table S1**). The control outgroup species were chosen because they comprise, along with *O. longistaminata*, all of the AA-genome *Oryza* species known to exist in Africa. Additionally, an *O. sativa*/*O. longistaminata* F_1_ hybrid from a controlled cross, two of its F_2_ progeny, and 14 recombinant inbred lines (RILs) were included in the study as controls for comparison with putative interspecific hybrids observed in the *O. longistaminata* accessions. The interspecific hybrid controls were not counted in the *O. longistaminata* sample size. The control interspecific individuals were from a cross between the Thai *O. sativa* ssp. *indica* cultivar RD-23 and a Nigerian *O. longistaminata* accession (Dayun and Sripichitt, 2000; Hu et al., 2003), both of which were included in the study. In total, 799 individuals were studied: 365 *O. longistaminata*, 309 *O. sativa*, 25 *O. barthii*, 83 *O. glaberrima*, and 17 known *O. sativa*/*O. longistaminata* hybrids or RILs. In a few accessions of all species, the species was mislabeled in the germplasm source, and sample sizes reflect the species identity observed in our study (**Table S1**). As discussed extensively, some genotypes labeled as *O. longistaminata* were putative interspecific hybrids with *O. sativa*; for simplicity, these are included in the *O. longistaminata* sample size (n = 365).

### Molecular markers

The materials were analyzed using 178,651 single-nucleotide polymorphisms (SNPs) and seven plastid microsatellite markers. DNA was extracted from lyophilized seedling-stage leaf tissue using the cetyltrimethylammonium bromide (CTAB) method with minor modifications for the samples sequenced in-house for this study (Fulton et al., 1995). To identify SNPs, restriction site-associated DNA sequencing (RAD-seq) libraries were prepared according to Clark et al. (2014) based on the method of Poland et al. (2012). In brief, DNA from each individual was digested with the restriction enzymes *Pst*I-HF and *Msp*I followed by ligation to barcoded adapters; then, the samples were pooled, and 200–500-bp fragments were selected and amplified by polymerase chain reaction (PCR). Libraries were sequenced on an Illumina Hi-Seq 2000 for 100-bp single-end reads at the Roy J. Carver Biotechnology Center at the University of Illinois. Demultiplexed reads were aligned to the Nipponbare IRGSP-1.0 reference genome using Bowtie 2.2.4 (Langmead et al., 2009). For all individuals in the study, SNPs were called with samtools 1.7 and bcftools 1.7. SNPs were filtered in vcftools 0.1.15 and TASSEL 5.2 such that at each site, only the two most common alleles of each variant were retained, the maximum proportion of heterozygous calls at a site was 0.5, and allelic read depths ranged from 7 to 400 (Bradbury et al., 2007). Individuals with >40% missing calls were dropped from further study. For each set of individuals used in a given iteration of population structure analysis, the SNPs were filtered to require a minimum minor allele frequency of 0.01 and a minimum site count equal to 66% of the number of individuals in the dataset.

Seven plastid markers were amplified from the DNA to check for consistency between maternal and nuclear genotypes, as well as to confirm the directionality of crosses that produced putative interspecific hybrids (**Table S2**). Some plastid markers were described by Ishii and McCouch (2000), and others were developed from data described by Yin et al. (2015) and Wambugu et al. (2015). The PCR products were size-separated by capillary electrophoresis with Genescan LIZ500 size standard on a 3730xL DNA Analyzer (Applied Biosystems, Foster City, CA, USA) at the Core DNA Sequencing Facility at the University of Illinois. Markers were scored with the software STRand 2.4.59.

### Genetic data analysis

To identify genetic subpopulations and assign individuals to those groups, two complementary analyses of population structure were conducted using the ADMIXTURE model in the software STRUCTURE 2.3.4 (Falush et al., 2003) and discriminant analysis of principal components (DAPC) in the R package adegenet (Jombart et al., 2010; Jombart and Ahmed, 2011). Three replications of ADMIXTURE at each K = 1 through K = 9 were run with a burn-in of 10,000 Markov chain Monte Carlo (MCMC) repetitions followed by 50,000 default MCMC repetitions, and the Evanno method as implemented by StructureHarvester was used to identify the optimal number of clusters (Earl and vonHoldt, 2012). For each individual, the proportion of the genome that originated from each cluster, or Q value, was reported from STRUCTURE (Pritchard et al., 2000). For DAPC, principal component analysis was first conducted with the *glPca* function, and then the *find.clusters* function was used to make initial groupings with the *n.start* option set to 500 to ensure convergence; *dapc* was used to assign individuals to each cluster. The number of clusters with the minimum Bayesian information criterion (BIC) was chosen as optimal. To distinguish putative interspecific hybridization of *O. longistaminata* from model noise or variation in individual relatedness to a shared common ancestor, putative *O. longistaminata* interspecific hybrids were defined to have 4.4%–95.6% admixture with control outgroups. The thresholds were chosen because the minimum admixture detected in control *O. sativa*/*O. longistaminata* RILs was 4.5% (**Table S1**). To further determine the ancestry of putative hybrids, three replications of STRUCTURE were run at K = 3 with the USEPOPINFO and PFROMPOPFLAGONLY options set to 1, MIGRPRIOR set to 0, and a burn-in of 10,000 MCMC repetitions followed by 50,000 default MCMC repetitions. Population information was specified only for individuals that had ancestry totally within one group (**Table S1**). Neighbor-joining trees were generated in the R package ape (Popescu et al., 2012) to observe local topologies. Genetic distance was calculated in TASSEL 5.2.30, and all trees were rooted using *root.phylo* in the R package ape at the *O. barthii* individual from accession SRR3231693, which had the maximum pairwise genetic distance observed. Geographic maps were drawn in ArcGIS 10.3.1 (ESRI). A plastid haplotype network was constructed with seven segregating microsatellites for 312 *O. longistaminata* individuals, 15 putative interspecific hybrids, 5 *O. sativa* individuals, and 3 *O. barthii* individuals with no missing data following Clark et al. (2014).

Spatial principal component analysis (sPCA) was conducted in the R package adegenet to identify spatial patterns in genetic variation. To reduce computation time, SNPs were thinned to a minimum distance of 100 bp, and only SNPs with a minor allele frequency greater than 0.01 that had been sampled in all individuals were used, yielding 3,974 SNPs. Genotypes were pooled by accession collection site, and allele frequency was estimated per collection site before analysis; a connectivity network of the collection sites was generated using the minimum spanning method. The lagged principal scores were interpolated by the natural neighbor method in ArcGIS 10.3.1.

Estimates of *F*_ST_ (genetic differentiation among subpopulations), *F*_IS_ (inbreeding coefficient), and *D* (Nei’s genetic diversity or expected heterozygosity) were calculated, adjusted for sample size following Nei and Chesser (1983), and averaged across loci using a custom R script. Pairwise estimates of genetic differentiation among genetic groups were estimated with Jost’s *D* (Jost, 2008) using the *pairwiseJostDnumeric* R function (Clark, 2016).

## Results

### Three *O. longistaminata* genetic groups and one interspecific hybrid group identified

**Figure 1:**
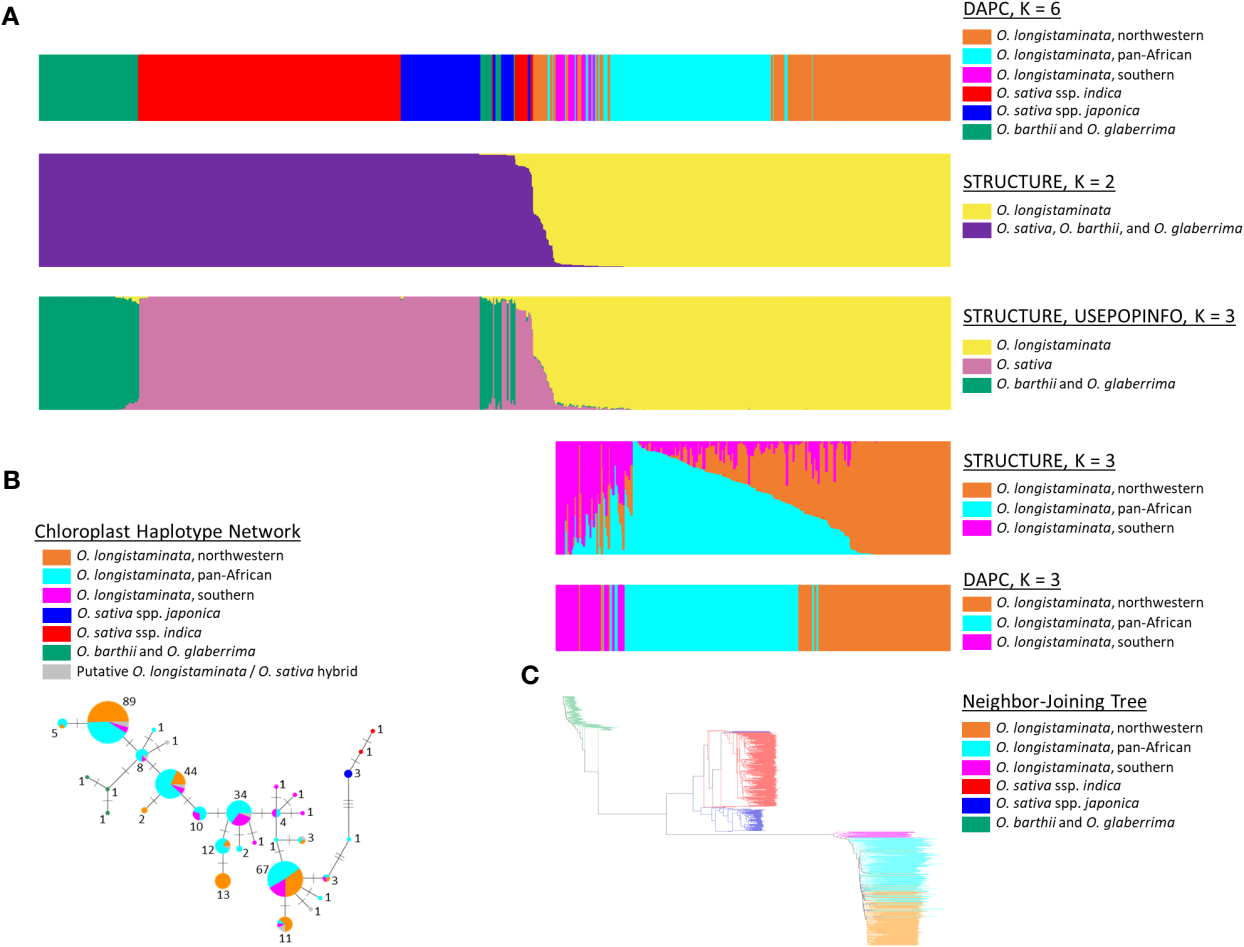
Population structure of *Oryza longistaminata* and phylogenetic relationships of *O. longistaminata* to AA-genome species *Oryza sativa*, *Oryza barthii*, and *Oryza glaberrima*. **(A)** Results for STRUCTURE, discriminant analysis of principal components (DAPC), and STRUCTURE with USEPOPINFO analyses of *O. longistaminata* (n = 346), *O. sativa* (n = 309), *O. barthii* (n = 25), *O. glaberrima* (n = 83), known *O. sativa*/*O. longistaminata* hybrids or recombinant inbred lines (RILs) (n = 17), and putative interspecific hybrids of *O. sativa* and *O. longistaminata* (n = 19). **(B)** Chloroplast haplotype network for 312 *O. longistaminata* individuals, 15 putative interspecific hybrids of *O. longistaminata* and *O. sativa*, 5 *O. sativa* individuals, and 3 *O. barthii* individuals. Each haplotype node is represented by a pie chart, the size of which is scaled to the number of individuals carrying the haplotype (shown adjacent) and the colors of which represent the proportion of individuals at that haplotype that carry a given nuclear genotype. **(C)** Neighbor-joining tree of all individuals studied except interspecific hybrids (both control and putative); individual edges are colored by DAPC-defined nuclear genotypes.

**Figure 2:**
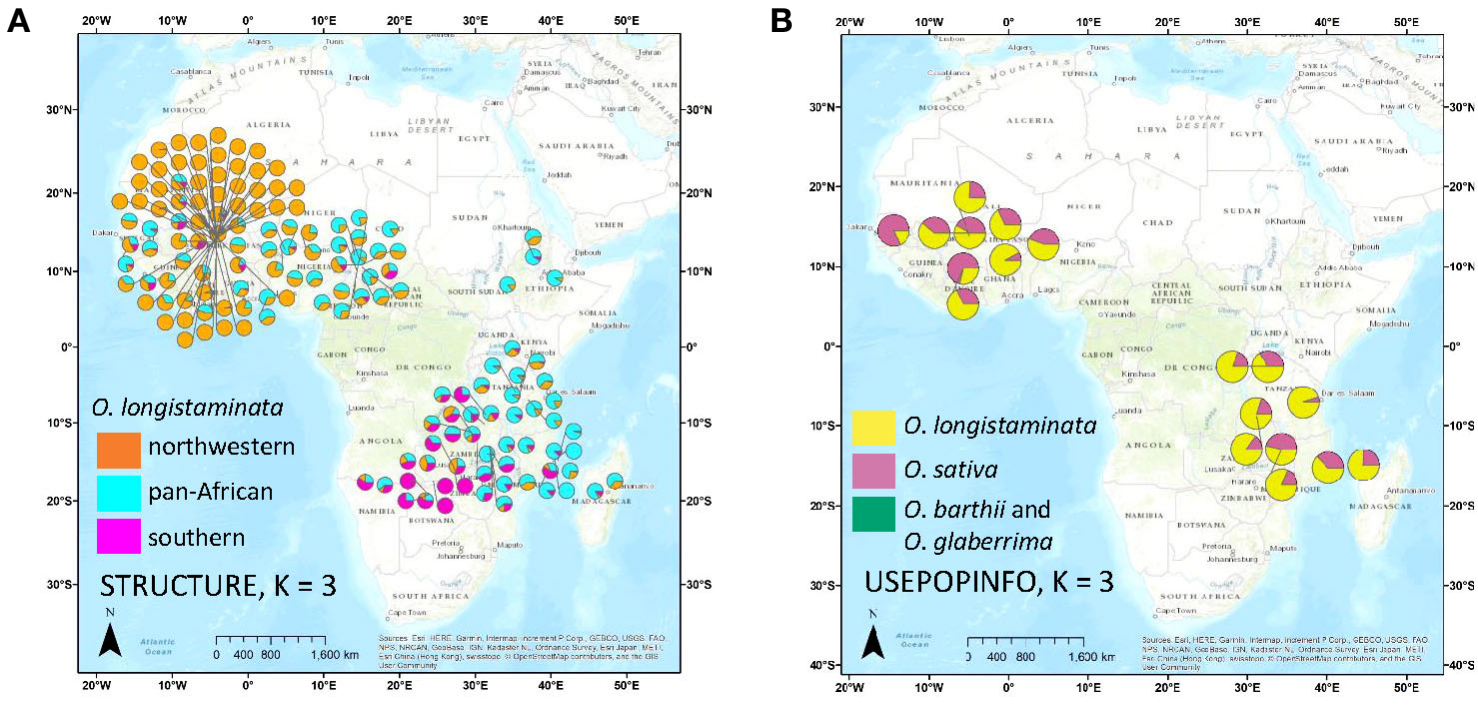
Maps of accession origins and STRUCTURE results for *Oryza longistaminata*. Ancestry of each accession is represented by a pie chart showing average Q for each genetic group for a given STRUCTURE run. **(A)** Ancestry at K = 3 of the *O. longistaminata* individuals <4.5% admixed with control outgroups, *Oryza sativa*, *Oryza barthii*, and *Oryza glaberrima*. **(B)** Ancestry as determined by STRUCTURE with USEPOPINFO of the *O. longistaminata* individuals >4.5% admixed with control outgroups.

*O. longistaminata* was differentiated from the control outgroups *O. sativa*, *O. barthii*, and *O. glaberrima* in the STRUCTURE and DAPC analyses (n_ind_ = 799, n_SNPs_ = 178,651; **Figure 1**). The DAPC analysis identified genetic groups with higher resolution, and the optimal number of groups was six: three groups of *O. longistaminata*, two groups of *O. sativa*, and one group comprising the African species *O. glaberrima* and *O. barthii* (**Figure 1A**). In contrast, K = 2 was optimal in the STRUCTURE analysis with one cluster including all *O. longistaminata* individuals and the other including all of the outgroup species, *O. sativa*, *O. barthii*, and *O. glaberrima* (**Figure 1A**). Notably, 19 individuals that had greater than 4.5% admixture with the outgroup species in STRUCTURE were putative recent interspecific hybrids (**Figures 1**, **2**; **Table S1**; **Table 1**). Many of the putative interspecific hybrids had phenotypes that were atypical for *O. longistaminata* (**Table S3**).

**Table 1:** Putative *Oryza sativa*/*Oryza longistaminata* progeny with >4.5% *O. sativa* ancestry and known F_1_, F_2_, and RIL control interspecific hybrids derived from a controlled cross.

Entry	Q values			Origin
	*O. sativa*	*O. longistaminata*	*O. barthii*–*O. glaberrima*	
Known *O. sativa*/*O. longistaminata* progeny from controlled crosses				
Bt136	0.46	0.54	0.00	F_2_ control
Bt135	0.47	0.53	0.00	F_1_ control
Bt137	0.49	0.51	0.00	F_2_ control
RILs15	0.82	0.18	0.00	RIL
RILs47	0.84	0.16	0.00	RIL
RILs31	0.86	0.14	0.00	RIL
RILs24	0.86	0.14	0.00	RIL
RILs60	0.88	0.12	0.00	RIL
RILs3	0.88	0.12	0.00	RIL
RILs43	0.88	0.12	0.00	RIL
RILs23	0.89	0.11	0.00	RIL
RILs27	0.89	0.11	0.00	RIL
RILs78	0.89	0.11	0.00	RIL
RILs11	0.89	0.11	0.00	RIL
RILs48	0.90	0.10	0.00	RIL
RILs42	0.90	0.10	0.00	RIL
RILs59	0.95	0.05	0.00	RIL
Putative recent hybrids: *O. longistaminata* with >4.5% *O. sativa* ancestry from IRRI genebank				
101741.002	0.79	0.18	0.04	Senegal
101211.001	0.71	0.29	0.00	Côte D’Ivoire
104300.002	0.46	0.53	0.02	Malawi
105075.002	0.44	0.55	0.01	Nigeria
101222.002	0.42	0.58	0.00	Mali
110404.002	0.42	0.56	0.02	India (Mali)
92650.002	0.39	0.61	0.01	Mali
89159.002	0.37	0.62	0.01	Mozambique
103886.001	0.34	0.66	0.00	Tanzania
106456.002	0.32	0.67	0.01	Mali
101211.002	0.32	0.68	0.00	Côte D’Ivoire
101222.001	0.24	0.76	0.00	Mali
89155.001	0.24	0.75	0.01	Mozambique
86480.002	0.20	0.80	0.00	Zambia
104300.001	0.19	0.81	0.00	Malawi
103886.002	0.19	0.81	0.00	Tanzania
86480.001	0.15	0.85	0.00	Zambia
105183.002	0.08	0.92	0.00	Ghana
101431.001	0.05	0.95	0.00	Tanzania

STRUCTURE was run at K = 3 with the USEPOPINFO and PFROMPFLAGONLY options on and MIGRPRIOR = 0. Q values are averaged over three replications. Predefined populations were *O. sativa*, *Oryza barthii–Oryza glaberrima*, and *O. longistaminata* with no interspecific ancestry.

RIL, recombinant inbred line; IRRI, International Rice Research Institute.

To investigate population structure within *O. longistaminata* without potential bias from outgroup species and putative interspecific hybrids, a second set of the DAPC and STRUCTURE analyses was conducted on only the *O. longistaminata* individuals with less than 4.5% interspecific admixture (n_ind_ = 351, n_SNPs_ = 75,371; **Figures 1A**, **2A**). The three *O. longistaminata* genetic groups observed in the prior analysis were again identified with DAPC (**Figure 1A**). Geographic maps showed that the three DAPC groups corresponded to Northwestern Africa, Pan-Africa, and Southern Africa (**Figure 2A**). Compared to the DAPC of all individuals, the DAPC of *O. longistaminata* with less than 4.5% interspecific admixture led to the reassignment of 25 individuals from the Pan-Africa group to the Southern Africa group (**Table S1**). The STRUCTURE analysis of the *O. longistaminata* subset identified K = 2 as optimal. Individuals without intraspecific admixture were concentrated in Northwestern Africa and Southern Africa, but most individuals were admixed between the two *O. longistaminata* groups. At STRUCTURE K = 3, all but five individuals in the Pan-Africa group were admixed intraspecifically with other *O. longistaminata* groups (**Figure 1A**; **Table S1**). At K = 3, the overall group membership of individuals did not change between DAPC and STRUCTURE (**Figure 1A**). The three *O. longistaminata* DAPC groups also formed distinct clades within the neighbor-joining tree (**Figure 1C**). An individual’s proportion of intraspecific admixture with each of the three *O. longistaminata* groups was moderately correlated with latitude (*r*^2^ = 0.37) and longitude (*r*^2^ = 0.44; **Table S1**).

The plastid haplotype network, which was constructed for *O. longistaminata*, the putative hybrids, and control *O. sativa* and *O. barthii*, consisted of 31 unique haplotypes (**Figure 1B**). The haplotype network was colored to indicate DAPC-defined groups based on nuclear genotypes (**Figure 1B**). As expected, plastid haplotypes did not overlap among *O. longistaminata*, *O. sativa*, and *O. barthii* (**Figure 1B**). The topology of the network differed from the neighbor-joining tree in that *O. longistaminata* fell between the two outgroups (**Figures 1B, C**). Among the *O. longistaminata* individuals that had less than 4.5% admixture with *O. sativa*, 25 unique haplotypes were identified. Some plastid haplotypes were more commonly found in a given *O. longistaminata* DAPC group, but no high-frequency haplotypes were specific to a nuclear genotype group. All of the putative recent interspecific hybrids had *O. longistaminata* plastid haplotypes (**Figure 1B**). In contrast, the control interspecific hybrids had an *O. sativa* plastid haplotype, as expected. The recent interspecific hybrids had nine of the 25 *O. longistaminata*-specific plastid haplotypes, with no particular overrepresentation of a single haplotype (**Figure 1B**).

**Figure 3:**
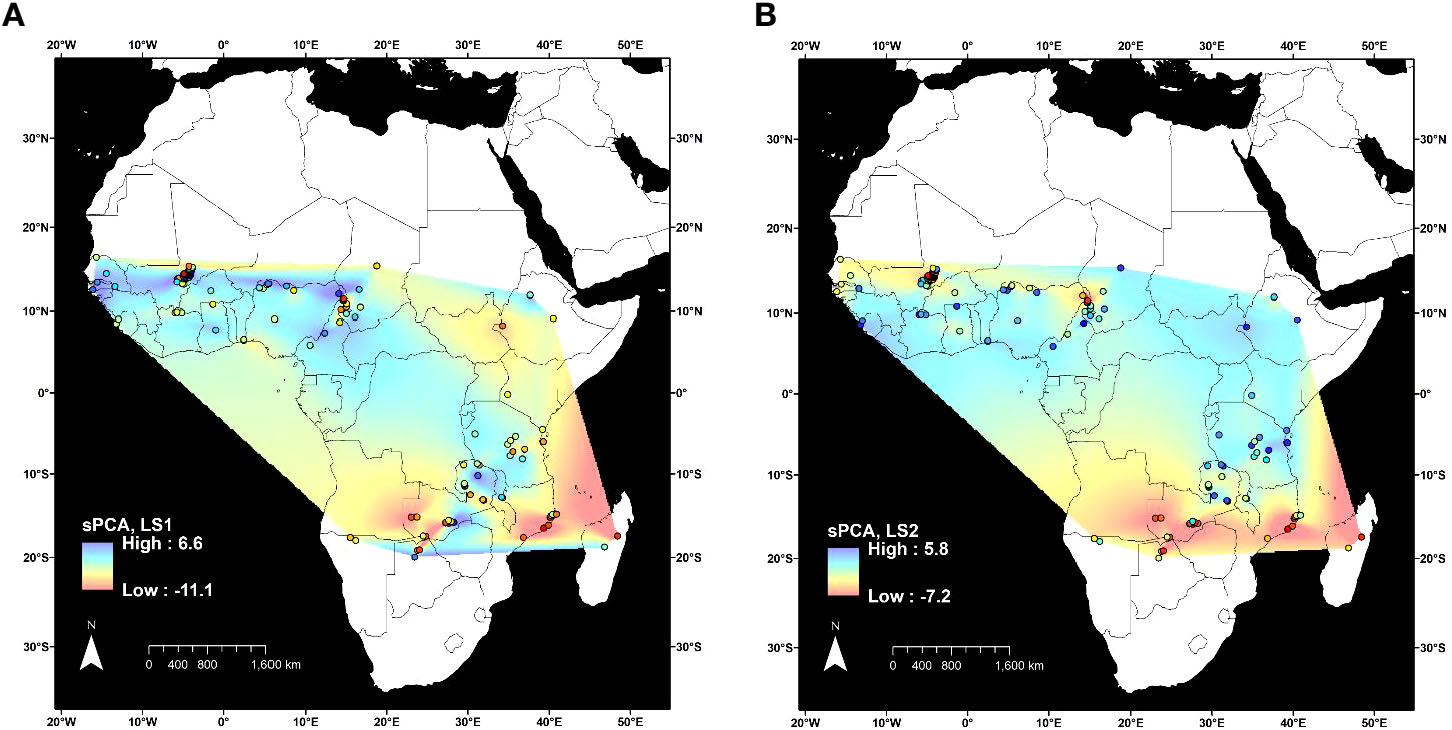
Spatial principal component analysis (sPCA) of 3,974 variants in 346 *Oryza longistaminata* individuals that were <4.5% admixed with control outgroups *Oryza sativa*, *Oryza glaberrima*, and *Oryza barthii*. Lagged principal component scores for two retained components were plotted for each collection site (circles outlined in black), and values were interpolated between sites by the natural neighbor method. **(A)** First lagged principal component score. **(B)** Second lagged principal component score.

Spatial principal component analysis of 3,974 SNPs at 153 geographic sites showed the overall geographic patterns of genetic structure observed with DAPC and STRUCTURE (**Figure 3**). Two eigenvectors were retained for analysis. The first eigenvector accounted for 11.3% of the genetic variation between sites and differentiated individuals in the northwest from all of the others (**Figure 3A**). The second eigenvector represented 3.1% of genetic variation between sites and differentiated individuals in the extreme south of the geographic distribution from all others (**Figure 3B**).

Because all but 16 of the 126 individuals in the Northwestern Africa *O. longistaminata* group were from a 64,000-km^2^ region of Mali, it is possible that high geographic sampling density led to the distinction of this group (**Figure 2A**). To examine the effects of uneven geographic sampling, STRUCTURE and DAPC were rerun using genetic data from a set of *O. longistaminata* accessions filtered to a minimum geographic distance of 25 km (n_ind_ = 173, n_SNPs_ = 74,793). STRUCTURE results still showed optimal K = 2 with individuals of singular group ancestry falling in Northwestern and Southern groups. However, DAPC indicated optimal K = 2, and the formerly distinct Northwestern group merged with the Pan-Africa group (data not shown). It is possible that denser geographic sampling of the entire species would maintain the Northwestern group and additionally reveal further substructure in other regions. For example, the sPCA suggested that there may be a distinct northeastern group despite sparse sampling from the northeast in the current dataset (**Figure 3B**).

Genetic diversity was similar among the three *O. longistaminata* DAPC groups, with the Northwestern Africa group having the lowest estimate of *D* (**Table 2**). Overall, genetic differentiation among the *O. longistaminata* groups, as indicated by *F*_ST_ and Jost’s *D*, was low (**Tables 2**, **3**). The Southern Africa group was the most diverse, the most differentiated from the other *O. longistaminata* groups, and also the most closely related to the outgroup species (**Tables 2**, **3**; **Figure 1C**). In contrast to the Southern Africa group’s high genetic diversity, the group’s inbreeding coefficient was approximately twice that observed for the other *O. longistaminata* groups (**Table 2**). The Pan-Africa subpopulation was the least differentiated from the whole. Among all pairwise comparisons of the *O. longistaminata* groups, the Northwestern Africa and Southern Africa groups were the most genetically differentiated (**Table 3**).

**Table 2:** Diversity statistics for *Oryza longistaminata* genetic groups identified by discriminant analysis of principal components (DAPC) based on 346 individuals that were <4.5% admixed with control outgroups.

Group	Number of SNPs	Number of individuals	*D*	*F*_ST_	*F*_IS_
Northwestern Africa	40,315	129	0.1331 ± 0.0007	0.0216 ± 0.0001	0.2805 ± 0.0013
Pan-Africa	50,061	174	0.1527 ± 0.0006	0.0127 ± 0.0000	0.2867 ± 0.0012
Southern Africa	41,038	43	0.1617 ± 0.0007	0.0232 ± 0.0002	0.4361 ± 0.0010
Africa, total	51,391	346	0.1568 ± 0.0006		

Statistics were calculated across 51,391 biallelic SNPs with depth > 7, MAF > 0.01, and site presence in at least 66% of individuals. The mean and standard error of each value are given across loci.

*D*, diversity (expected heterozygosity); *F*_ST_, subpopulation differentiation from the total population; *F*_IS_, inbreeding coefficient; SNPs, single-nucleotide polymorphisms; MAF, minor allele frequency.

**Table 3:** Pairwise Jost’s *D* statistic showing differentiation between *Oryza longistaminata* genetic groups identified with discriminant analysis of principal components (DAPC) based on 346 individuals that were <4.5% admixed with control outgroups.

Group	Pan-Africa	Southern Africa
Northwestern Africa	0.0142 ± 0.0002	0.0383 ± 0.0004
Pan-Africa		0.0203 ± 0.0002

Statistics were calculated using 51,391 biallelic SNPs with depth > 7, MAF > 0.01, and site presence in at least 75% of individuals. The mean and standard error of each value are given across loci.

SNPs, single-nucleotide polymorphisms; MAF, minor allele frequency.

### *O. sativa*/*O. longistaminata* hybrids and bidirectional introgression

To further investigate the ancestry of the previously identified putative recent interspecific hybrids (>4.5% interspecific admixture) and the *O. longistaminata* observed to have a low proportion of interspecific admixture (<4.5%), a STRUCTURE analysis was conducted with USEPOPINFO (n_ind_ = 799, n_SNPs_ = 178,651; **Figure 1A**). Individuals with zero interspecific admixture were assigned to three predefined groups—*O. sativa*, *O. glaberrima*–*O. barthii*, and *O. longistaminata*—and used to assign ancestry from those groups to other individuals (**Table S1**). As controls, the analysis included a known *O. sativa*/*O. longistaminata* F_1_ hybrid from a controlled cross, two of its F_2_ progeny, and 14 RILs. As expected, the known interspecific hybrids from controlled crosses were correctly identified as hybrids of *O. sativa* and *O. longistaminata* (**Table 1**). The analysis indicated that all individuals previously observed to have more than 4.5% interspecific admixture had ancestry predominantly from *O. sativa* (Asian) and *O. longistaminata* (African) and negligible ancestry from *O. barthii* (African) and *O. glaberrima* (African; **Figure 1A**, **Table 1**). Ancestry from *O. sativa* was 70% or higher in two individuals (**Table 1**), indicating introgression of *O. longistaminata* genes into Asian domesticated rice; however, higher ancestry from *O. longistaminata* than *O. sativa* was observed in the other 17 interspecific individuals, indicating introgression of genes from the domesticated Asian species into the undomesticated African species. Four individuals had Q values similar to the F_1_ and F_2_ controls, and several other individuals had ancestry ratios near those expected in the BC_1_ and BC_2_ generation for introgression into both *O. longistaminata* and *O. sativa* (**Table 1**). The putative early-generation hybrids between *O. sativa* and *O. longistaminata* may have been from recent crossing events and were from areas of sub-Saharan Africa where *O. sativa* is currently cultivated (Food and Agriculture Organization of the United Nations, 2023).

Notably, the results from STRUCTURE with the USEPOPINFO option indicated that all of the individuals in the Southern Africa *O. longistaminata* group had low-level admixture with *O. sativa* (mean = 3.7%; range, 2.4%–5.3%) and also with *O. barthii*–*O. glaberrima* (mean = 1.3%; range, 0.8%–2.0%). Not all individuals showing interspecific admixture belonged to the Southern group. However, *O. sativa* ancestry of less than 4.5% was rare in the Northwestern Africa individuals (mean, 0.0%; range, 0.0%–2.5%) and Pan-Africa individuals (mean, 0.4%; range, 0.0%–3.8%). Ancestry from *O. barthii*–*O. glaberrima* was also rare in Northwestern Africa (mean, 0.0%; range, 0.0%–0.7%) and Pan-Africa (mean = 0.1%, range = 0.0%–1.5%) *O. longistaminata* groups.

## Discussion

Our study adds support to the current literature consensus that *O. longistaminata* is more closely related to the Asian species *O. sativa* than the African species *O. barthii* and *O. glaberrima*. We report for the first time that there appear to be three main genetic subpopulations in the sampled *O. longistaminata*, with populations primarily structured by geographic distance. Moreover, we identified recent spontaneous interspecific hybrids of *O. sativa* and *O. longistaminata* in the IRRI genebank collection, which can be immediately useful for breeding. A further novel and notable contribution of this study was the observation of low-level admixture between *O. sativa* and *O. longistaminata* in Southern Africa only, which was likely the result of ancient hybridization upon the introduction of *O. sativa* to Madagascar approximately 1,000 years before the present. Additionally, we provided new nuclear and chloroplast markers for the species.

### Relationship between *O. longistaminata* and the other AA-genome species in Africa

Our study indicated that the undomesticated African rice, *O. longistaminata*, shared a more recent common ancestor with Asian domesticated rice, *O. sativa*, than the African *O. glaberrima*–*O. barthii* group had (**Figure 1C**). Most previously conducted studies, including those with the highest genome coverages and sample sizes, have concluded that *O. longistaminata* is more closely related to *O. sativa* than to *O. barthii* and *O. glaberrima* (Wambugu et al., 2015; Zhu et al., 2014; Cheng et al., 2002; Ren et al., 2003; Kwon et al., 2005; Duan et al., 2007; Marathi et al., 2015); only two studies found *O. longistaminata* to be more closely related to *O. barthii* and *O. glaberrima* than *O. sativa* (Park et al., 2003; Yin et al., 2015). Our study adds support to the current consensus because it uses the highest genome coverage and the greatest number of individuals of any study to date. A more recent divergence between *O. longistaminata* and its Asian relative *O. sativa* than its African relatives, *O. glaberrima* and *O. barthii*, suggests that Africa has two distinct lineages of native AA-genome rice species, likely associated with independent migration events.

### Population structure in *O. longistaminata*

To our knowledge, this is the first study to evaluate the *O. longistaminata* population structure across most of sub-Saharan Africa using densely spaced genome-wide molecular markers. Three genetic groups of *O. longistaminata* were identified (**Figure 1A**), two of which were associated with distinct geographic regions of Africa (Northwestern and Southern; **Figure 2A**), which will be useful information for conserving germplasm of this species and for using this wild relative to improve domesticated rice. Though differentiation among the three *O. longistaminata* populations was low, geographic distance appeared to be the main factor associated with genetic differentiation; sharp barriers to gene flow were not observed (**Figure 3**). STRUCTURE, DAPC, sPCA, and Jost’s *D* consistently indicated that the extremes of differentiation in *O. longistaminata* were between the Northwestern and Southern populations (**Figures 1**, **2**; **Table 3**). Similarly, the individuals of the Pan-Africa group showed a gradient of admixture with each of the other genetic groups roughly according to their geographic proximity to each other. These observations were consistent with the species’ biology, which includes perennation with dispersal of rhizomes along rivers during floods (Kleynhans et al., 2007; Green and El-Moghraby, 2009), obligate outcrossing due to self-incompatibility (Ghesquière, 1986), dispersal of seed by birds (Gupta, 2004), and adaptation to tropical environments that were relatively stable during periods of glaciation (Maley, 1996).

The mechanisms of genetic differentiation within *O. longistaminata* likely included drift and perhaps isolation associated with differences in flowering time. Populations at the edges of a species’ geographic range, such as the Northwestern and Southern populations, can have low population densities that leave individuals with few neighbors with which to outcross; over time, lower effective population size accelerates genetic drift and increases inbreeding. Consistent with this scenario, the Southern Africa *O. longistaminata* group had substantially greater inbreeding than the other groups, though it also had unexpectedly high genetic diversity (**Table 2**). Given that the Northwestern and Southern populations are separated by more than 20 degrees of latitude and the equator, variation in flowering time could also lead to isolation. Variation in flowering time could be due to genetic differences in day-length sensitivity, the timing of the growing season, or a combination of both. *O. longistaminata* has been previously observed to be a short-day plant, though some individuals (most commonly observed near domesticated rice fields) are insensitive to photoperiod (Ghesquière, 1986). In the Urbana, IL greenhouse (40°N), most accessions flowered only during the short days of late autumn and winter, with substantial differences in flowering time among accessions; however, some flowered during the long days of summer (i.e., were apparently day-neutral). Similarly, *O. longistaminata* accessions collected in Ethiopia (8–12°N) were observed to flower during long days at Jinghong Rice Breeding Station in China (20°N; G. Melaku, Addis Ababa University, Ethiopia, pers. comm.).

### Origins of the Southern Africa *O. longistaminata* group

The Southern Africa group was unique among the three *O. longistaminata* groups in that all individuals had low-level estimated admixture with *O. sativa* (**Figure 1**; **Table S1**). Two hypotheses were proposed to account for the apparent interspecific admixture that is a defining feature of the Southern Africa group: 1) this group is more similar to the ancestral *O. longistaminata* population than the other groups, and the apparent admixture with *O. sativa* is an artifact that represents alleles in common to the ancestral *O. longistaminata* population and its Asian AA-genome relatives, with many of the alleles subsequently lost in the more derived Pan-Africa and Northwestern Africa groups; 2) the low level of admixture with *O. sativa* in the Southern Africa *O. longistaminata* group was the result of ancient interspecific hybridizations and subsequent introgression. However, these hypotheses are not mutually exclusive: it is also possible that the Southern Africa *O. longistaminata* group could be the center of origin for this species and was also the first to subsequently hybridize with *O. sativa*.

The proximity of the Southern Africa group to the outgroup species in the neighbor-joining tree could be consistent with the first hypothesis of greater similarity to the ancestral *O. longistaminata* population, but it would also be expected if the admixture was actually the result of interspecific hybridization (**Figure 1C**). Additionally, the ordered decrease in genetic diversity of the three *O. longistaminata* groups from Southern Africa to Pan-Africa to Northwestern Africa could indicate that the species radiated north and west from a southern center of diversity; however, introgression of alleles from another species could explain the high diversity observed in the Southern group (**Table 2**). The Southern Africa group individuals not only had low levels of ancestry with *O. sativa* but also had low levels of ancestry with the *O. barthii*–*O. glaberrima* group (**Figure 1**; **Table S1**). It would have been unlikely that the Southern Africa group individuals hybridized with both *O. sativa* and *O. glaberrima*, given that *O. glaberrima* is primarily cultivated in Northwestern Africa and that *O. barthii* is sympatric with all of the *O. longistaminata* groups identified (Vaughan, 1994). Thus, the ordered increase in apparent *O. barthii*–*O. glaberrima* ancestry from the posited basal Southern group to the intermediate Pan-Africa group to the most derived Northwestern group would be consistent with an ordered decline in the populations’ relatedness to the AA-genome common ancestral population. This could suggest that apparent coancestry with both *O. sativa* and *O. barthii*–*O. glaberrima* is due to residual relatedness to a common ancestor. However, the observed low-level admixture with *O. barthii*–*O. glaberrima* in the Southern Africa *O. longistaminata* group was also similar to levels observed in the 19 recent hybrids between *O. sativa* and *O. longistaminata* (>4.5% admixture) regardless of their geographic origin (**Figure 1**), which indicates that this likely represents baseline relatedness of *O. barthii*–*O. glaberrima* and *O. sativa* to a common ancestor. Thus, the common-ancestry hypothesis would not account for the greater observed admixture with *O. sativa* (mean = 3.7%; range, 2.4%–5.3%) than *O. barthii*–*O. glaberrima* (mean = 1.3%; range, 0.8%–2.0%) in the Southern Africa *O. longistaminata* group. Instead, we would expect many *O. longistaminata* individuals that are introgressed with *O. sativa* to also show a lower level of coancestry with *O. barthii*–*O. glaberrima*, representing alleles derived from a common ancestor.

Bolstering the interspecific hybridization hypothesis is the observation that the current pattern of low-level *O. sativa* admixture in *O. longistaminata* (ubiquitous in the Southern Africa group but rare in the Northwestern Africa and Pan-Africa groups) mirrors the historical timing and location of Asian domesticated rice cultivation in Africa. Intriguingly, recent archaeological and molecular genetic data indicate that *O. sativa* was introduced to southeastern Africa via Madagascar and the Comoros Islands by farmers who migrated from Southeast Asia across the Indian Ocean as early as ~1,000 years before present (Mather et al., 2010; Crowther et al., 2016), which could account for a low level of ancient interspecific admixture in the current Southern Africa *O. longistaminata* group. However, in Northwestern Africa, *O. glaberrima* was domesticated from the indigenous wild *O. barthii* ~3,000 years ago (Linares, 2002). Though Asian domesticated rice, *O. sativa*, was likely introduced into West Africa by Europeans as early as the mid-16th century, extensive production of Asian rice in West Africa and its replacement of African rice cultivars primarily occurred recently, in the second half of the 20th century (Linares, 2002). Thus, opportunities for hybridization between *O. sativa* and *O. longistaminata* in West Africa were likely infrequent until modern times. However, the distribution of *O. glaberrima* was limited to West Africa. Thus, in southeastern Africa, the introduction of *O. sativa* directly from Asia ~1,000 years ago (Mather et al., 2010; Nayar, 2014; Crowther et al., 2016) would not have faced competition for cropping space from pre-existing domesticated rice. The archeological record indicates that these ancient rice-growing farmer-migrants from Asia to southeast Africa maintained the knowledge and practice of growing Asian domesticated rice in their new home, as many of their descendants in Madagascar still do today. Given that our study (**Figure 2**) and others (Chu and Oka, 1970b; Kanya, 2010; Kilewa, 2014) have documented with molecular genetic data recent bidirectional introgression between *O. sativa* and *O. longistaminata*, throughout the latter’s natural geographic range (i.e., not limited to Southern Africa), then it would be reasonable to expect that similar ancient sympatric populations would have also produced interspecific progenies where they existed. Moreover, the ancient hybridization hypothesis is consistent with both the high genetic diversity and high inbreeding estimates observed for the Southern Africa *O. longistaminata* group because introgression of alleles from another species would be expected to increase genetic diversity, and *O. sativa*/*O. longistaminata* F_1_ hybrids are often self-compatible (Ghesquière, 1986; Hu et al., 2003). High rates of inbreeding would not typically be expected if the population represented a center of origin and diversity. Thus, the data from our study suggest that interspecific hybridization and introgression in Northwestern Africa are predominantly recent, whereas in Southern Africa, both recent and likely ancient introgressions have occurred. If the ancient hybridization hypothesis is correct, then the Pan-Africa group may be the most genetically similar to the ancestral *O. longistaminata* population because it is genetically diverse yet relatively free of low-level introgressions from *O. sativa*.

### Implications of spontaneous *O. sativa*/*O. longistaminata* hybridization and bidirectional introgression for germplasm conservation and breeding

The current study and at least three prior studies (Chu and Oka, 1970b; Kanya, 2010; Kilewa, 2014) have produced molecular evidence of recent spontaneous hybridizations between *O. sativa* and *O. longistaminata*, lending credence to studies that documented interspecific hybrids without confirmation with molecular markers (Bezançon et al., 1977; Ghesquière, 1986; Kiambi et al., 2018; Kiambi et al., 2005). Moreover, spontaneous progeny derived from backcrosses to each of the parent species have also been observed (**Table 1**). In contrast to findings of spontaneous interspecific hybridization, plant breeders have typically regarded the cross between *O. sativa* and *O. longistaminata* as exceptionally difficult; to rescue interspecific progeny from the abortive endosperm, 1- to 2-week-old embryos or ovules were cultured *in vitro* (Ramos et al., 2016; Dayun and Sripichitt, 2000). The current study identified 19 early-generation interspecific progeny (>4.5% admixed) from Côte D’Ivoire, Mali, Nigeria, Tanzania, Mozambique, Malawi, and Zambia out of 365 individuals with *O. longistaminata* ancestry (**Table 1**). This discovery rate of 5.2% was approximately double the ~2.5% maximum reported from controlled crosses thus far (Kaushal and Ravi, 1998). The present study cannot eliminate the possibility that the recent hybridization events that led to the observed interspecific progeny occurred during seed increases of *O. longistaminata* accessions in the IRRI screenhouse, but even if so, the rate of hybrid recovery was exceptional compared to that of controlled crosses. The greater frequency of interspecific progeny backcrossed to *O. longistaminata* than to *O. sativa* observed in this study could be explained by observational bias because interspecific individuals with greater ancestry from *O. sativa* would be less likely to exhibit phenotypes typical of *O. longistaminata*, and therefore explorers or germplasm curators may have been less likely to sample or retain them. However, it is also possible that backcrosses to *O. sativa* are typically less fit (e.g., exhibit hybrid breakdown) than backcrosses to *O. longistaminata*. Consistent with the trends observed here, Kilewa (2014) reported that in Tanzania, the spontaneous production of F_1_ interspecific hybrids from *O. sativa* fields where wild *O. longistaminata* grew sympatrically averaged 7.3% for *O. longistaminata*/*O. sativa* crosses and 2.6% for *O. sativa*/*O. longistaminata* crosses. Additionally, Sacks et al. (2006) observed that seed-set for crosses between an *O. longistaminata*/*O. sativa* F_1_ backcrossed to *O. sativa* varied among 11 cultivar backcross parents. Given that few controlled crosses between *O. sativa* and *O. longistaminata* have been attempted, it is possible that there are substantial differences in general and/or specific interspecific crossability within one or both parental species that could be used to advantage by plant breeders to facilitate introgression for rice improvement.

Although targeted introgression of genes from *O. longistaminata* into domesticated Asian rice may be desirable in breeding, the introgression of genes from domesticated Asian rice into wild populations of the African *O. longistaminata* may be considered undesirable genetic pollution (Maxted and Guarino, 2006). Data from our study indicate that interspecific hybridization and introgression in Northwestern Africa are predominantly recent, whereas in Southern Africa, both recent and ancient introgressions have likely occurred. If historical regional differences in the timing of Asian rice cultivation explain why ancient introgressions of *O. sativa* genes into wild *O. longistaminata* are common in populations from Southern Africa but not from West Africa, then in the future, the West Africa populations of *O. longistaminata* will likely accumulate a greater proportion of genes from *O. sativa* than currently observed.

Given that obtaining *O. sativa*/*O. longistaminata* F_1_ progeny from controlled crosses has required considerable effort (Causse and Ghesquiere, 1991; Khush et al., 1991; Dayun and Sripichitt, 2000; Ramos, IRRI, Philippines, pers. comm.; Kaushal and Ravi, 1998; Ramos et al., 2016), which has been a major barrier to using *O. longistaminata* for rice improvement, the 19 early-generation interspecific progenies identified in the current study are a valuable resource for breeding improved cultivars of domesticated Asian rice. It should also be possible to mine additional genotypes with *O. sativa* introgressions from *O. longistaminata* germplasm collections, thus greatly facilitating breeding. Armed with this knowledge, including which *O. longistaminata* accessions in the IRRI genebank harbor interspecific hybrids with *O. sativa* (**Tables 1**, **S1**), rice breeders should take full advantage of this newly discovered opportunity. Furthermore, introgression efficiency from controlled crosses can likely be improved by screening *O. longistaminata* and *O. sativa* germplasm—including close relatives of the observed interspecific hybrids—for interspecific crossability to identify individuals with relatively high general crossability to the other species. Moreover, among the natural interspecific hybrids in the IRRI genebank may be individuals with exceptional crossability to both parental species, *O. sativa* and *O. longistaminata*, and would thus be valuable bridging lines. Given the high genetic and geographic diversity of *O. longistaminata*, along with its well-documented biotic stress tolerance (Thottappilly and Rossel, 1993; Soriano et al., 1999; Rakotomalala, 2001; Gupta, 2004; Du, 2008; Panigrahi and Rajamani, 2008), likely abiotic stress tolerance (Liu et al., 2004; Giuliani et al., 2013; Atwell et al., 2014), and other known traits of agronomic value (Marathi et al., 2015; Gichuhi et al., 2016; Reinhold-Hurek et al., 2015; Brar, 2005; Ramos et al., 2016), we expect that further selective introgression of *O. longistaminata* genes into *O. sativa* will result in new cultivars of great value to humanity.

## Data availability statement

The datasets presented in this study can be found in online repositories. The names of the repository/repositories and accession number(s) can be found in the article/**Supplementary Material**.

## Author contributions

ML: Data curation, Formal Analysis, Investigation, Visualization, Writing – original draft. LC: Supervision, Data curation, Writing – review & editing. SZ: Resources, Writing – review & editing, Investigation. FH: Resources, Writing – review & editing, Supervision. DT: Resources, Writing – review & editing. RH: Resources, Supervision, Writing – review & editing. ES: Conceptualization, Funding acquisition, Project administration, Resources, Supervision, Writing – original draft.
