# Supplementary material for: Solving the mystery of Obake rice in Africa: population structure analyses of Oryza longistaminata reveal three genetic groups and evidence of both recent and ancient introgression with O. sativa
Source: Front Plant Sci. 2023 Nov 15;14:1278196. doi: 10.3389/fpls.2023.1278196 (PMC10684938; doi:10.3389/fpls.2023.1278196)
Supplement: Supplementary file 3 [file Table_3.docx]

| SUPPLEMENTARY TABLE 2 Atypical phenotypes in individuals from *O. longistaminata* accessions, such as lack of rhizomes, short stature, short stamens, and filled grains produced by selfing. Images of the phenotype are provided where available, or presence of the phenotype is noted. Lack of recorded atypical phenotype does not imply normal phenotype. Not all interspecific hybrids had an atypical phenotype, consistent with reports by Kanya (2010) and Kilewa (2014). | | | | | |
| --- | --- | --- | --- | --- | --- |
| **Study_sampleid** | **Lack of rhizomes** | **Short stature** | | **Short stamens; filled grains produced by selfing** | |
| 83826.001 | 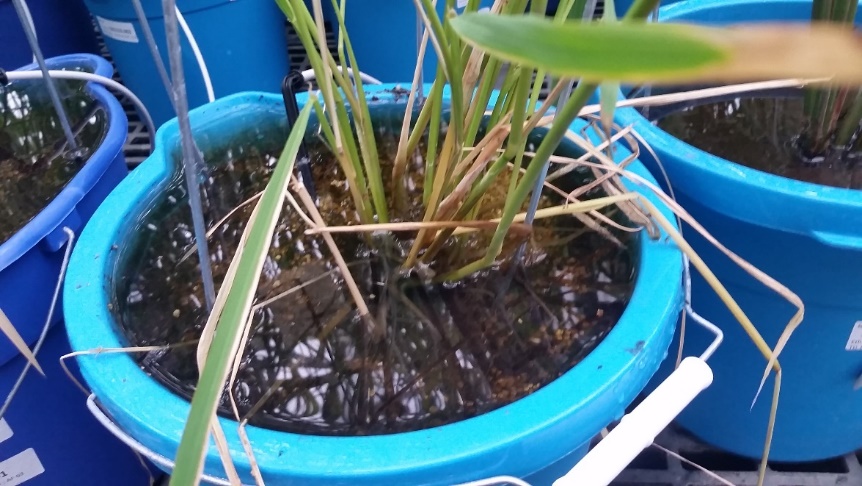 | | 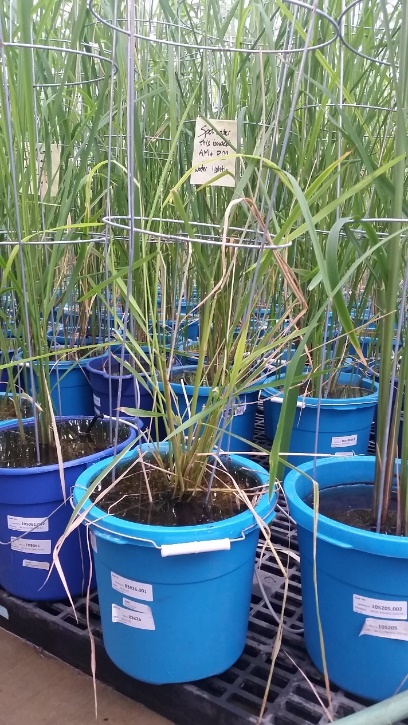 | | 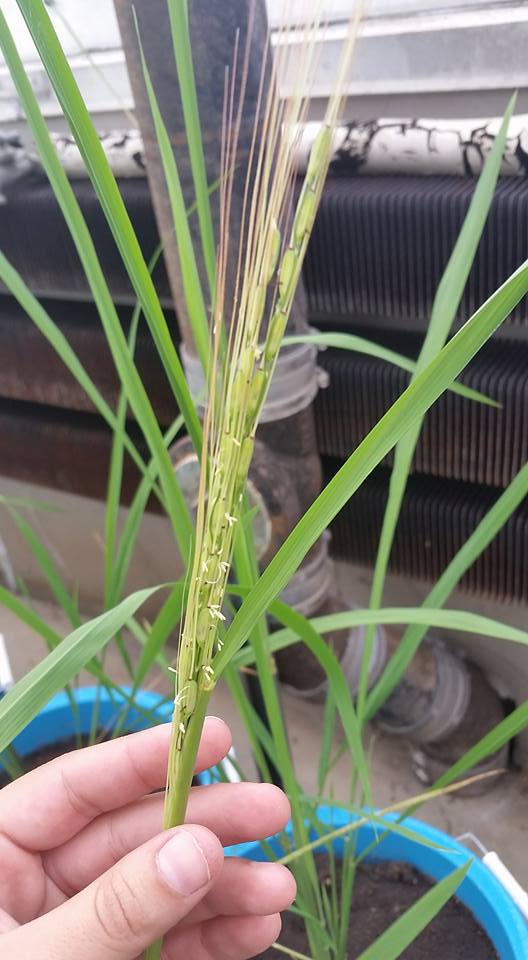 |
| 83826.002 | 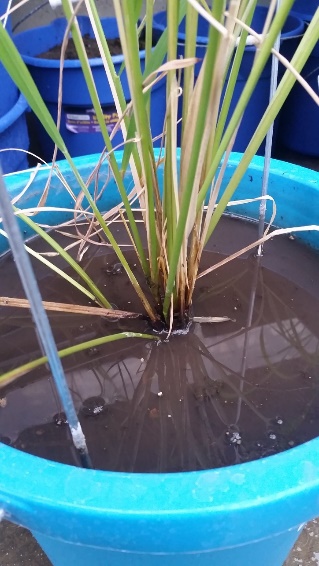 | | 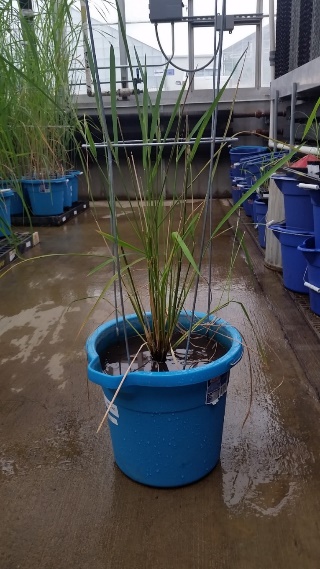 | | 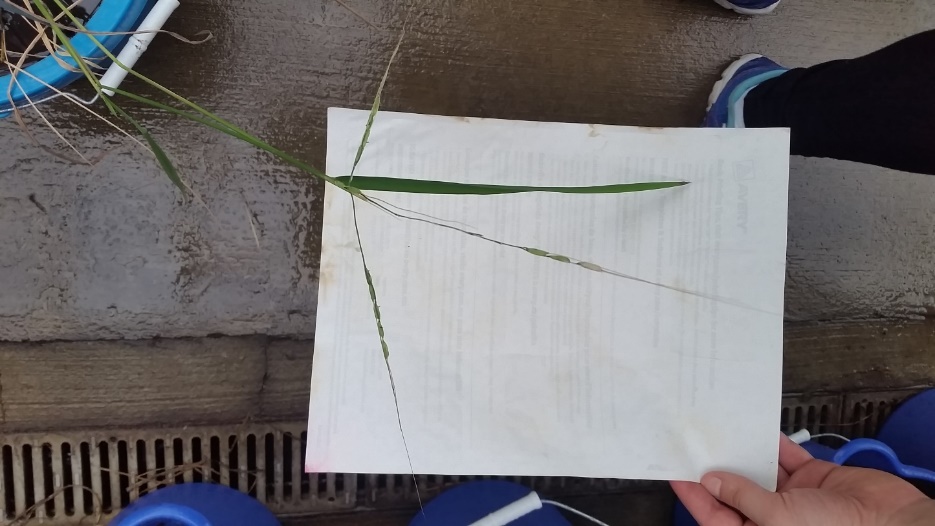 |
| 100930.001 | 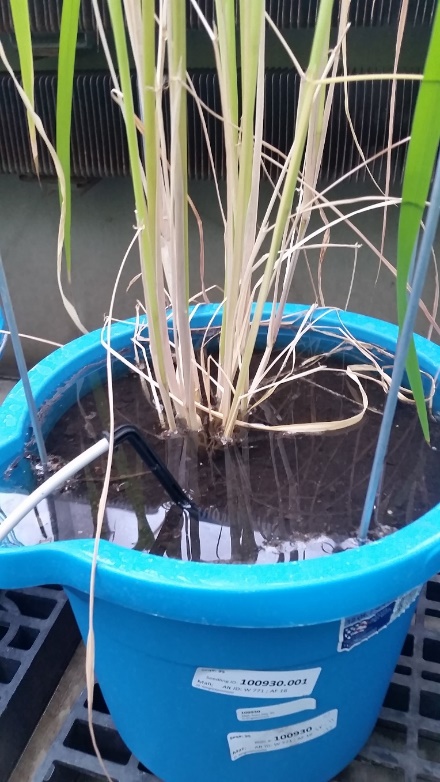 | | NA | | 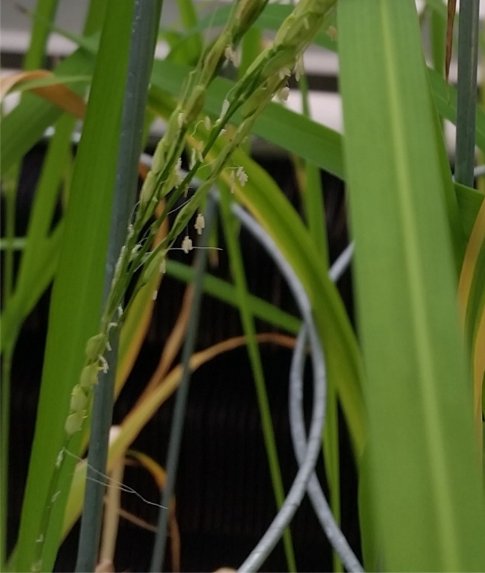 |
| 100930.002 | 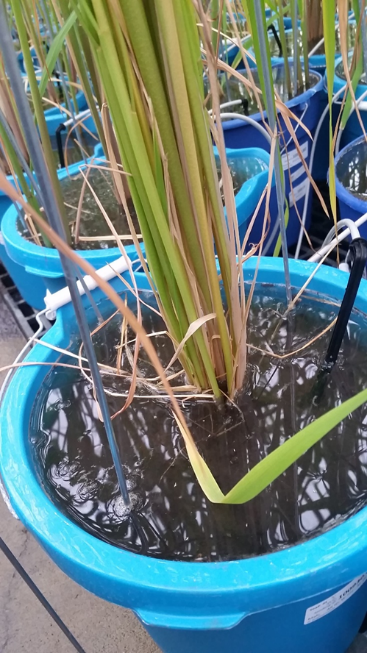 | | NA | | 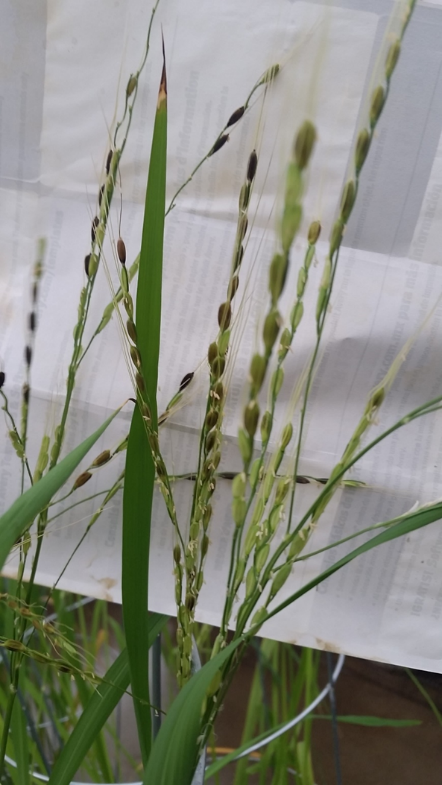 |
| 86483.002 | 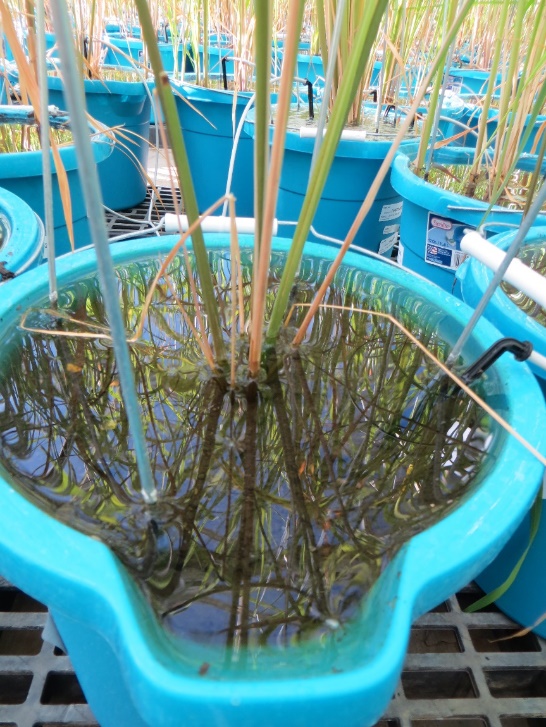 | | NA | | NA |
| 104300.002 | 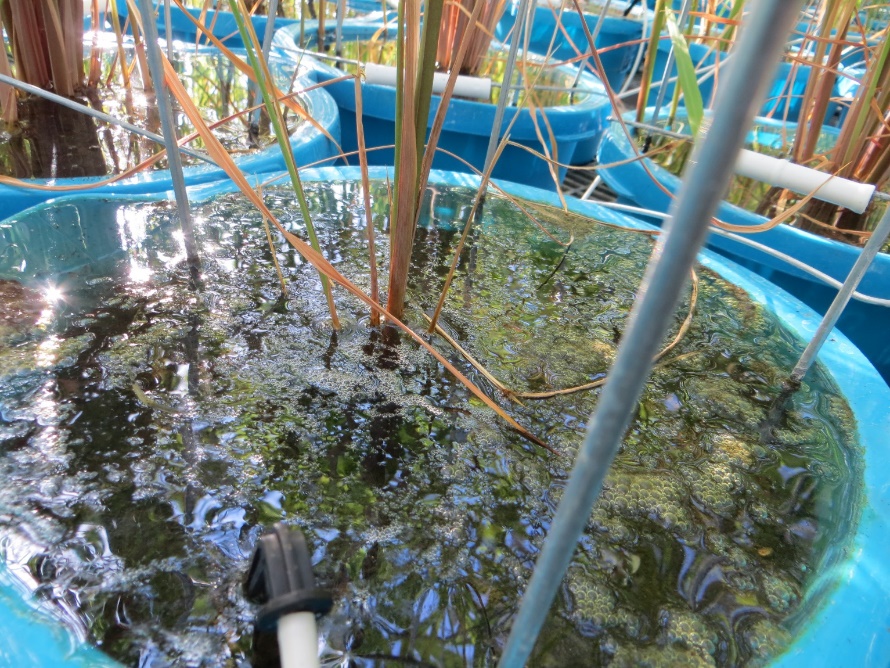 | | NA | | NA |
| 103886.001 | 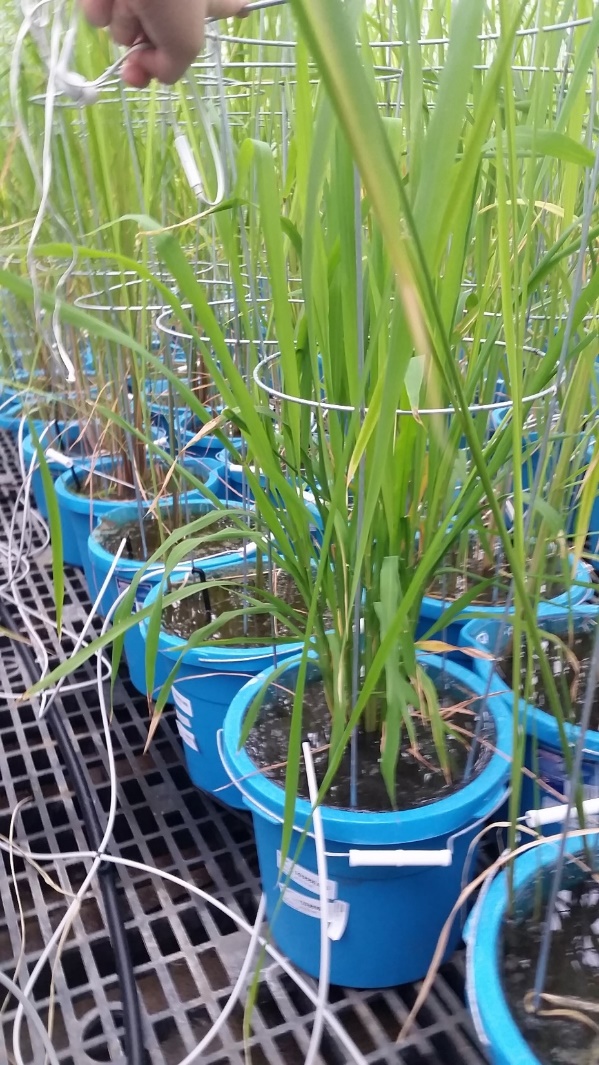 | | 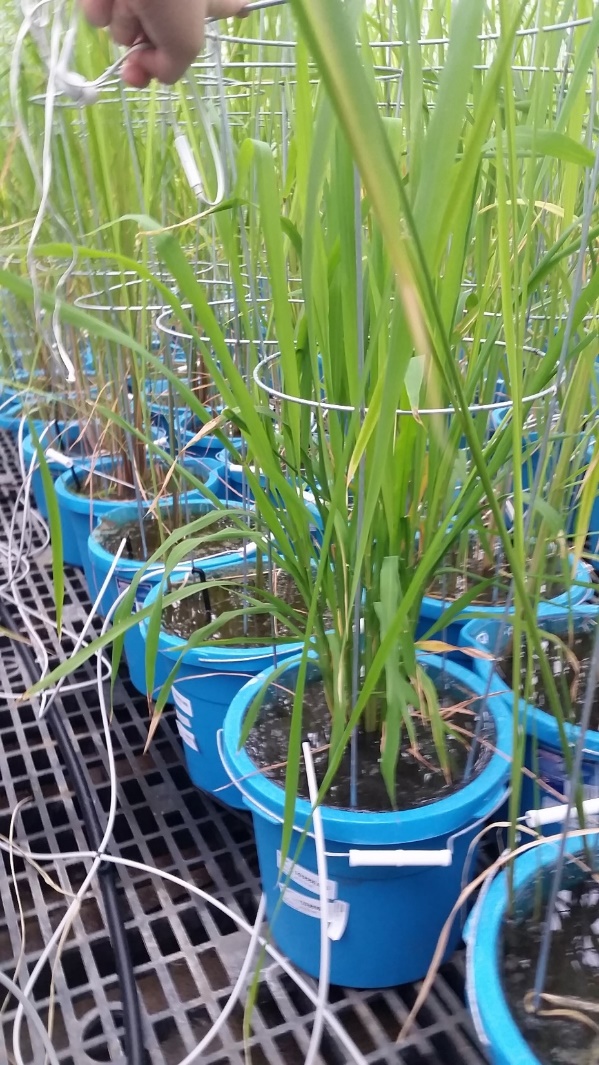 | | NA |
| 101211.001 | 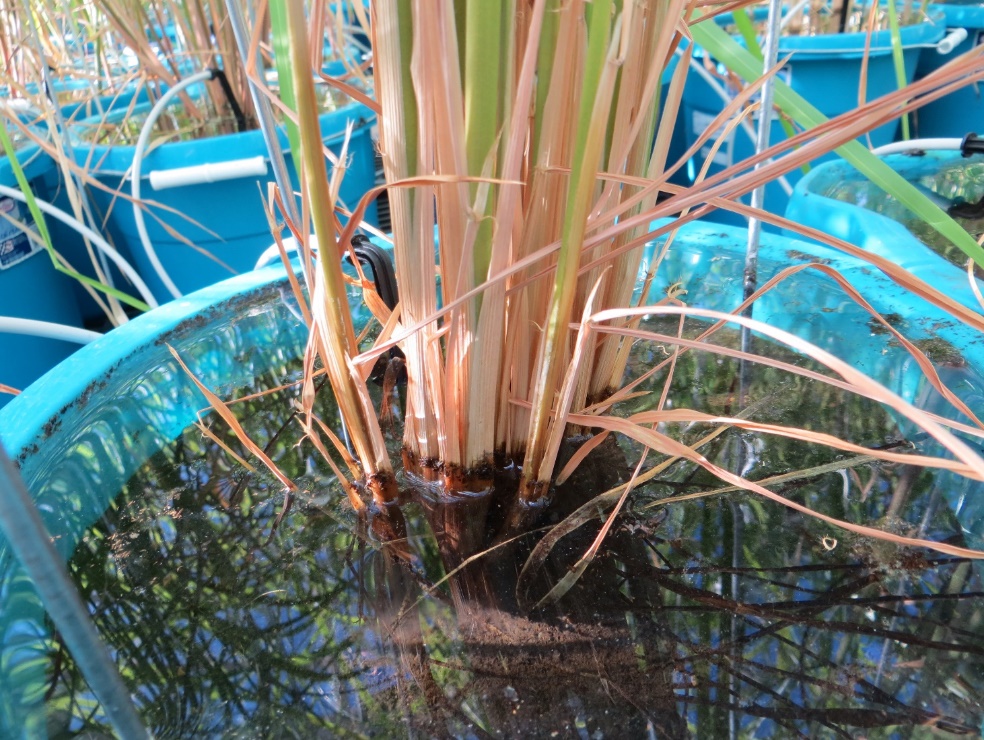 | | NA | | NA |
| 101211.002 | 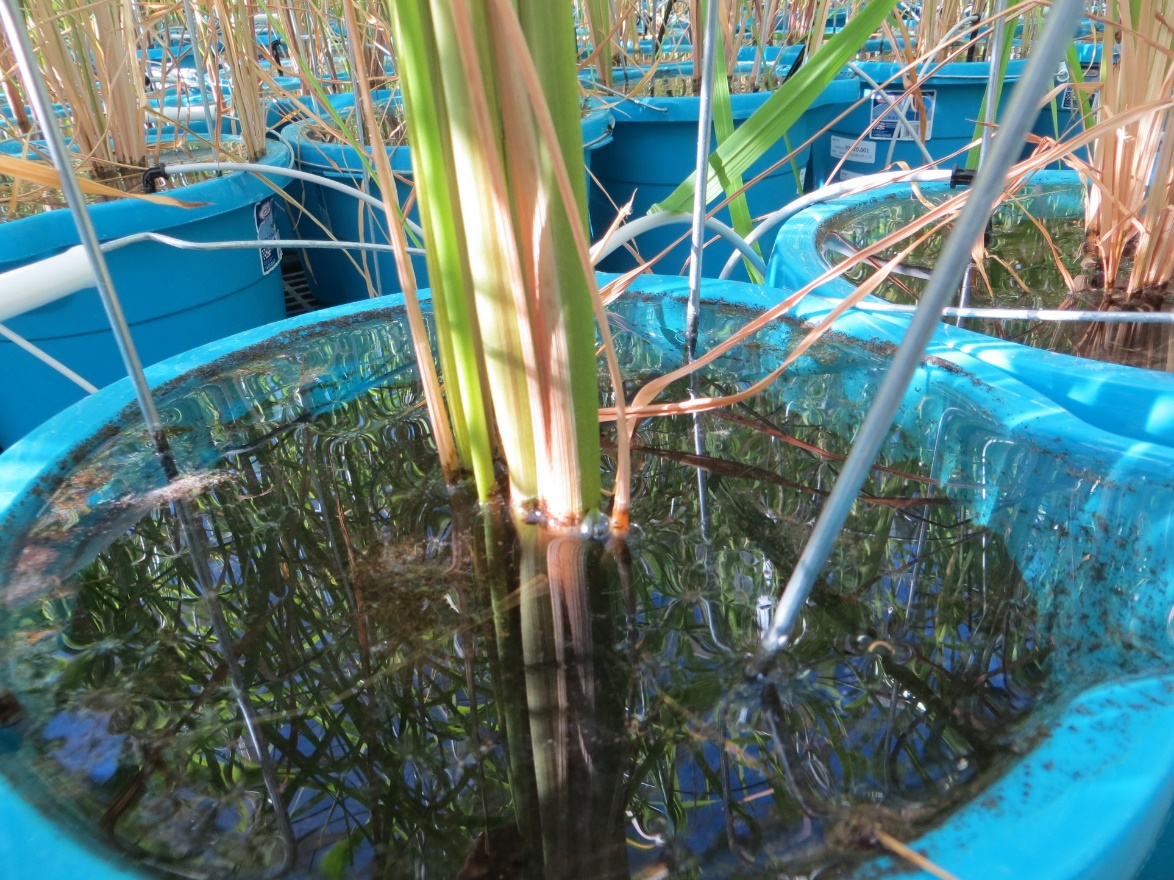 | | NA | | NA |
| 101436.001 | 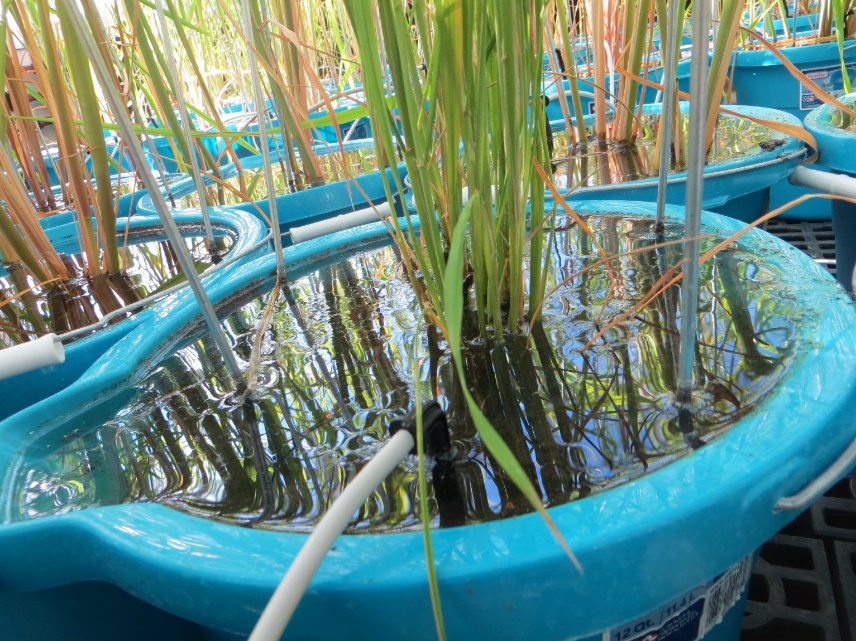 | | NA | | NA |
| 101222.002 | 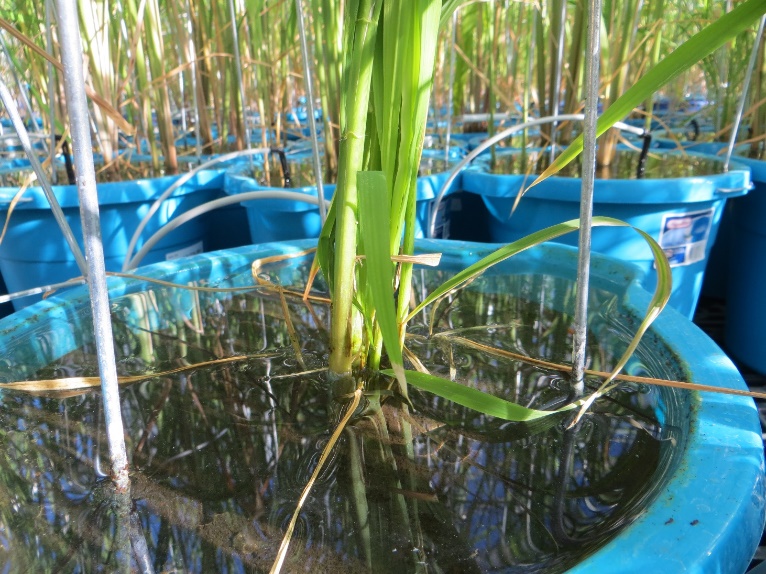 | | Short stature noted | | NA |
| 101741.002 | 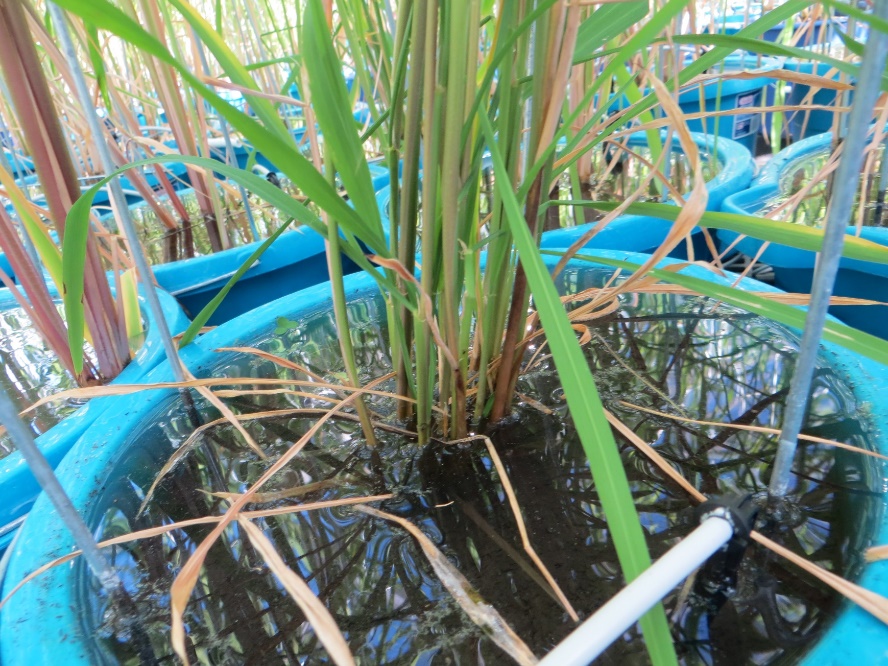 | | Short stature noted | | NA |
